# Speech–Brain Frequency Entrainment of Dyslexia with and without Phonological Deficits

**DOI:** 10.3390/brainsci10120920

**Published:** 2020-11-28

**Authors:** Juliana Dushanova, Yordanka Lalova, Antoaneta Kalonkina, Stefan Tsokov

**Affiliations:** 1 Institute of Neurobiology, Bulgarian Academy of Sciences, 1113 Sofia, Bulgaria; mightynerd@gmail.com; 2 Institute of Population and Human Studies, 1113 Sofia, Bulgaria; j_lalova@doctor.bg; 3 Centre for Speech Therapy, Ministry of Education and Science, 1000 Sofia, Bulgaria; akalonkina@abv.bg

**Keywords:** developmental dyslexia, auditory cortex, inferior frontal cortex, frequency oscillations

## Abstract

Developmental dyslexia is a cognitive disorder characterized by difficulties in linguistic processing. Our purpose is to distinguish subtypes of developmental dyslexia by the level of speech–EEG frequency entrainment (δ: 1–4; β: 12.5–22.5; γ1: 25–35; and γ2: 35–80 Hz) in word/pseudoword auditory discrimination. Depending on the type of disabilities, dyslexics can divide into two subtypes—with less pronounced phonological deficits (NoPhoDys—visual dyslexia) and with more pronounced ones (PhoDys—phonological dyslexia). For correctly recognized stimuli, the δ-entrainment is significantly worse in dyslexic children compared to controls at a level of speech prosody and syllabic analysis. Controls and NoPhoDys show a stronger δ-entrainment in the left-hemispheric auditory cortex (AC), anterior temporal lobe (ATL), frontal, and motor cortices than PhoDys. Dyslexic subgroups concerning normolexics have a deficit of δ-entrainment in the left ATL, inferior frontal gyrus (IFG), and the right AC. PhoDys has higher δ-entrainment in the posterior part of adjacent STS regions than NoPhoDys. Insufficient low-frequency β changes over the IFG, the inferior parietal lobe of PhoDys compared to NoPhoDys correspond to their worse phonological short-term memory. Left-dominant 30 Hz-entrainment for normolexics to phonemic frequencies characterizes the right AC, adjacent regions to superior temporal sulcus of dyslexics. The pronounced 40 Hz-entrainment in PhoDys than the other groups suggest a hearing “reassembly” and a poor phonological working memory. Shifting up to higher-frequency γ-entrainment in the AC of NoPhoDys can lead to verbal memory deficits. Different patterns of cortical reorganization based on the left or right hemisphere lead to differential dyslexic profiles.

## 1. Introduction

Children with developmental dyslexia have difficulties in acquiring reading and writing skills despite their normal scores on intelligence tests [1,2]. Theories, giving different concepts of developmental dyslexia, attempt to explain the variety of linguistic and cognitive deficits. According to the “dual route” model, the words can be read either by the lexical or sublexical route [3]. In the lexical route, the words are directly recognized as lexicon members and associated with verbal semantic representations, when they are familiar, automatically identified by their visual form [3]. In the sublexical route, based on grapheme to phoneme correspondence rules for unfamiliar words, the word is broken down into its constituent letters and corresponding phonemes [3]. Impairment in either of these routes will result in a characteristic pattern of reading difficulties [4]. When the children’s deficits are in phonological skills (so-called phonological dyslexia [5]), they probably use the lexical route to compensate for the sublexical route [6], and naming irregular words well, but not pseudo-words. When the problems are in the lexical route, the sublexical route is used [7]. Then, the pseudo-words could be processed, but not the irregular words (surface dyslexia [8,9]). These deficits, including either a phonological one [1,2,3,4,5,6,7,8,9,10,11,12,13,14], visual magnocellular deficit [15,16], or all of them accompanied by attentional deficits, can occur in dyslexia not only independently of one other but together in combined deficits [11,17,18]. They are selectively associated with low accuracy or with slow performance on reading subskills [19], problems with clearly seeing letters and their order, orienting and focusing of visual–spatial attention [20,21,22]. The deficits in the magnocellular pathway were associated with letter decoding disability [23,24,25]. The magnocellular system is the visual input to the dorsal pathway that mediates motion perception and object localization [26] due to the projections to the visual motion-sensitive area and the posterior parietal cortex. For reading, the dorsal pathway has a major role in directing visual attention and control of eye movements [20,21]. Generally, due to anterior and middle temporal lobe deficits, affecting the primary ventral, occipitotemporal, and lexical route, children with severe phonological disabilities rely more on ventral (occipitotemporal lexical [5]) brain regions, whereas other dyslexic readers with less pronounced phonological deficits use more the dorsal (occipitoparietal sublexical) route. According to the dual-route model for speech processing [27], the ventral stream involved anterior, superior, and middle portions of the temporal lobe in processing speech signals for comprehension. The dorsal pathway with a beginning on the boundary of the temporal and parietal lobes involves the posterior planum temporale and posterior frontal lobe in translating acoustic speech signals into articulatory representations, encompassed the posterior parts of left inferior temporal and inferior frontal gyri, and the premotor and motor cortices [27]. The ventral stream is bilaterally organized, while the dorsal stream is strongly left-dominant [27]. Therefore, neuropsychological evidence for dyslexic subgroups supposes that partially distinct neural substrates as a dorsal and a ventral route, respectively, underlie sublexical and lexical processes [7,21]. A lexicosemantic route involves the left basal temporal language area, the posterior part of the middle temporal gyrus, and the inferior frontal gyrus [28]. A sublexical route involves left-lateralized superior temporal area, supramarginal gyrus, and the opercular part of inferior frontal gyrus [28]. Nevertheless, controversies still exist about the anatomical substrates involved in lexical and sublexical reading routes [28,29].

According to the double deficit hypothesis [30] that the phonological deficits and the naming speed deficits are separate sources of reading disability, categorized two dissociated single-deficit subgroups and one subgroup with combined deficits. The naming-speed deficit subtype has significant problems in the rapid recognition and retrieval of visually presented linguistic stimuli, timed reading, and showing accuracy and latency problems in word identification. The phonological subtype has difficulties in the phonological tasks that hamper the ability to segment the phonemes in reading and/or processing of speech sounds, in indicating phonological blending, word attack [30].

Based on neuroscience research, the neuronal mechanisms underlying the different cognitive dyslexic subtypes with and without phonological deficits [17] treat the phonological processes as separate, specific sources of disability in developmental dyslexia [2].

In different language studies, the dyslexics exhibited impaired access to phonological representations because of dysfunctional connectivity between the auditory cortices and the left inferior frontal gyrus [31]. They also showed a neural hypoactivation in the left ventral occipitotemporal area, the inferior temporal, inferior frontal gyrus, and in the temporoparietal cortex [32,33,34,35,36]. A study [35] of phonological awareness and reorientation of attention found various left and right frontal effects on brain activation. Such atypical neural mechanisms confirm that the phonological deficit in dyslexia is not necessarily a result of the disturbed magnocellular function [2,35]. Increased activation in the left inferior frontal gyrus of children with dyslexia has been observed to compensate for their dysfunction in the left posterior regions (posterior part of superior temporal gyrus, the supramarginal, and angular gyri of the left inferior parietal lobe). To obtain a better understanding of the neurobiological basis of dyslexia and the heterogeneity in brain activation profiles of dyslexia, it is important to identify the neural differences between dyslexics with predominant phonological deficits and those with less pronounced ones [2,16,35,37].

Systematically atypical neural activity, associated with auditory processing, has been observed in children with developmental dyslexia in EEG research [38,39,40,41,42]. Effective auditory processing is the basis for the accurate encoding of the phonological structure of words. The EEG oscillatory entrainment (synchronization) to the speech rhythms in delta (1–4 Hz), theta (4–8 Hz), beta (15–30 Hz), and gamma (>30 Hz) frequencies underlies the speech encoding [43,44]. To perceive words accurately, listeners can rely mainly on the retained information about the envelopes of the strongest speech characteristics as syllables (4 Hz) and phonemes (35 Hz) preferentially by the cortical delta/theta and low-frequency gamma oscillations [38,45,46,47,48]. The auditory sampling in the delta/theta [45,47] and the low-frequency gamma range can be altered during the early stage of processing in the auditory cortical areas of dyslexics [39,49]. The delta-beta frequency relations in the auditory cortex and motor brain areas are the basis for temporal predictive precision [50]. In dyslexia, the syllabic sampling at the delta frequency rate may be altered [38,49,51] or the phonemic sampling at the low gamma frequency range may be too slow or too fast compared to controls, which has direct consequences for phonological processing. Slower phonemic sampling can reduce phonemic discrimination [52], while the too rapid sampling of oscillations can load the auditory system with over-detailed time-frequency information, saturating both the delta acoustic capacity [53] and the phonological working memory. The contribution of theta frequency synchronization is not significant enough to clarify the underlying deficits in phonemic representations in dyslexia [54]. The delta frequency modulation at the stressed syllable’s rate (2 Hz) might occupy a more prominent role than the theta range [39,48]. Some authors [54] claim that the increase in the neural synchronization to phoneme rates in dyslexia correlates with later shown phonological and reading skills that might be attributable to specific experience-driven changes in the auditory cortex. Other authors show an increase in the neural synchronization to phoneme rates in controls [39].

In many studies of dyslexia, researchers use artificial and manipulated stimuli or continuous texts [43,44,48,49,50,54,55,56], and only a few studies apply paradigms with sublexical speech sounds or words [2,20,26]. In the case of studies with words, the effect of language comprehension arises from the word meanings. Difficulties of dyslexics exist in identifying word stimuli, demonstrating different interactions between abnormal sensory processes and higher-order processes, leading to prosodic processing dysfunction in developmental dyslexia. In our study, the auditory stimuli are natural pronounced words and pseudowords on the Bulgarian language, which belongs to the family of shallow orthographies. The elongated pronunciations of words and the dynamic stress of more words give them a different meaning. The voice stimuli with these language’s oddities contain rich prosodic information, which facilitates the semantic processing (comprehension) even in single words, unlike a vocal-synthesized speech signal.

We hypothesize that the stimulus-brain alignment arguably results from auditory and motor tuning throughout the speech and language comprehension, allowing auditory research to contribute to the issue of how the neuronal oscillatory activity reflects linguistic and cognitive deficits in dyslexia. The first goal is that the entrainment profile in the phonological-related frequency bands as delta, beta, and gamma frequencies could differentiate children with dyslexia from controls. The second goal of the current study is to give a neuro-functional view of the heterogeneity of different dyslexic types, based on auditory neural speech–EEG frequency entrainment.

## 2. Materials and Methods

### 2.1. Subjects

An evaluation of the reading ability of 980 children from 11 primary schools (8–9 years old) in Sofia, Bulgaria, found 120 children with a common learning disability. After an additional neuropsychological screening [57] sixty children were chosen to participate in a longitudinal behavior study. The children underwent a series of tests, a DDE-2 (Battery for the Evaluation of Developmental dyslexia and Dysorthography [58] for Bulgarian children [59]), psychometric tests for the evaluation of phonological awareness, tests for the evaluation of reading and writing skills [60], and Raven’s Progressive Matrices test for nonverbal intelligence [61]. Twenty-six children were found with developmental dyslexia. Reliable electrophysiological data were obtained from forty-six children: 26 children with developmental dyslexia (17 boys and 9 girls) and 21 age-matched normal children (12 boys and 8 girls). The age range for both groups was 8–9 years old. All children and their parents gave their informed consent for inclusion before they participated in the study (Supplementary Files 1 and 2). The study was conducted according to the Declaration of Helsinki, and the protocol was approved by the Ethics Committee of Institute of Neurobiology and the Institute for Population and Human Studies, BAS (approval No. 02-41/12.07.2019) and the State Logopedic Center, and the Ministry of Education and Science (approval No. 09-69/14.03.2017). All participants in the study spoke Bulgarian as their first language. All children were right-handed. The handedness was assessed by a classification of hand preference [62]. The participants had non-verbal intelligence scores of 98 or higher [62]. All children passed an audiometric test to confirm that they have normal hearing. The controls were paid for participating.

### 2.2. Procedure

A standardized test battery DDE-2 was utilized to assess a reading (112 words/48 nonwords with increasing complexity; 10 homonyms—choosing the correct meaning of the word from 4 variants; search for misspellings of 32 words) and writing (dictation of 47 words/26 nonwords with increasing complexity; 12 sentences). The results are presented in standard scores of reading time/speed and accuracy (Table S1).

The standardized for Bulgarian children test battery “Reading abilities” [60] comprised two phonological tasks, each with 10 words, reading aloud of a text with 133 words and dictation of 30 sentences filling in the missing compound word. In the first phonological task (“without the first sound-letter”), it was tested the ability of the child first to identify and then to miss the first sound of the heard word in his/her answer. In the other phonological task (“without the last syllable”), it was tested the ability of the child to fragmentize the word into syllables and to miss the last syllable of the word in his/her answer. The execution time and the number of the correct answers were scored (Table S1). According to the test batteries, the dyslexic children showed lower scores compared to the control group (Table S1).

### 2.3. Speech Paradigm

All subjects had pure-tone hearing thresholds better than a 20-dB hearing level in both ears at standard audiometric frequencies between 250 Hz and 8 kHz. The participants were required to discriminate auditory stimuli (words/pseudowords) presented in pseudo-random order. The words were with natural semantic content (mean duration of 0.701 ± 0.115 ms, mean modal frequency 1.460 ± 0.216 Hz), while the pseudowords derived from the words by replacing all the vowels (mean duration 0.727 ± 0.126 ms and mean modal frequency 1.413 ± 0.237 Hz). The words were selected according to the child age with low and high-frequency use and encompassed different parts of the speech (nouns, adjectives, verbs, numerals, prepositions, adverbs, pronouns, and conjunctions; Supplementary File 3). The Frequency Dictionary is created based on a hundred thousand Bulgarian words from conversational language (the 100,000 words were divided into 5 subgroups of 20,000 words each). According to the frequency of use, each word is assigned a certain rank. The smaller the rank, the more often the word is used. The numerator in the frequency indicates how often a particular word appears in the whole text group. While the denominator shows the number of subgroups in which this word appears—the maximum number is 5 [63]. The words with a high frequency were from 387/5 to 138/5 and a corresponding rank of 46 to 110.5, while those with an average frequency of 88/5 to 40/5 and a rank of 150 to 273, and the words with a low frequency—from 13/3 to 25/5 and rank from 368.5 to 624.5. The words in the task had no orthographic and phonological neighborhoods.

To preserve the original vocal and prosodic features, a native Bulgarian child spoke the words and pseudowords. Recordings were made in a professional recording studio with a sampling rate of 44 kHz. Sound Blaster speakers were used for the audio-presentation of the stimuli at a comfortable sound level (65 dB). The stimuli (20 words/20 pseudowords) were presented binaurally at an interstimulus interval (ISI) of 1.5–2.5 s in two to four blocks during daily experimental sessions or 80 experimental trials. Participants were asked to blink only during ISI to prevent artifacts during stimuli. The participants were instructed to push a button with the right hand when hearing a word and to push a different button with the left hand when the stimulus was a pseudoword. Participants were evaluated on their ability to correctly identify words/pseudowords. Reaction times and performance accuracy were compared between groups and conditions using a Kruskal–Wallis nonparametric test (KW test, *p* < 0.05).

### 2.4. Data Analysis

#### 2.4.1. EEG Preprocessing

The electroencephalogram (EEG) was recorded with an in-house developed 40-channel Wi-Fi EEG system (offline cut-off frequencies of 0.1–70 Hz) using dry EEG sensors (each sensor consists of 16 golden pins arranged in a star-shaped configuration, Brain Rhythm Inc., Taiwan). Reference sensors were placed to both processus mastoidei and rereferencing to global median reference was performed online to eliminate the volume conduction [64]. A soft ground sensor was placed on the forehead. The sensors were positioned on the head according to the international 10–20 system: F3-4, C3-4, T7-8, P3-4, O1-2; Fz, Cz, Pz, and Oz; and additional positions according to the 10–10 system: AF3-4, F7-8, FT9-10, FC3-4, FC5-6, C1-2, C5-6, CP1-2, CP3-4, TP7-8, P7-8, PO3-04, and PO7-08. The skin impedance was controlled to be less than 5 kΩ. The sampling EEG rate was 250 Hz and a second-order notch filter was applied to remove the power-line noise. The reaction time was determined automatically through the reaction time box. EEG trials contaminated with eye blinks and muscle activities were removed (±200 µV). Only trials with correct responses were included in the analysis. A criterion for a signal-to-noise ratio (SNR) was applied for each trial. The SNR is the peak-to-peak amplitude of the mean signal divided by twice the standard deviation of the noise. The noise is obtained by subtracting the average of each trial. For a sensor, the noise is simply a collection of the residuals obtained when the average signal is subtracted from each trial, and the standard deviation is the one obtained for this trial set. Only those trials that meet the removal noise criteria were included further in the analysis. After artifact rejection, the mean number of trials per condition was 30 correct trials over a subject. The subject with the smallest number of artifact-free and correct trials provided 20 segments per condition.

#### 2.4.2. Coherence Analysis

The audio stimuli were downsampling to 250 Hz, time-locked to EEG signals. The audio stimuli and the EEG data were segmented into 900-ms-long epochs. A magnitude-squared coherence was determined over the frequency rhythms (1 ÷ 80 Hz) of each correctly recognized stimulus and its concomitant EEG trial for each possible combination of EEG sensors, frequencies, and subjects (mscohere MATLAB function). The magnitude-squared coherence estimate is a function of frequency with values between 0 and 1. These values indicate how well two signals at each frequency correspond. The magnitude-squared coherence is a function of the power spectral densities of each sound stimulus and its corresponding EEG trial, and their cross power spectral density [65], using Welch’s overlapped averaged periodogram method [66,67]. The periodogram uses a 50% overlapping Hamming window, converting the signal from the time- to frequency-domain. The power spectrum is divided into bins with a step of 0.5 Hz. The Welch method uses a discrete signal corresponding to the EEG frequency components at each frequency bin. The word discrimination was assessed in each condition as the percentage of correctly recognized words. It was computed by dividing the number of correctly recognized words after pooling across trials, normalized by the number of presented words. The speech encoding was examined by speech–EEG frequency entrainments at multiple temporal rates in delta (δ, 2–4 Hz), beta (β, 12.5–22 Hz), low gamma1 (γ1, 25–35 Hz), and high gamma2 (γ1, 35–80 Hz) range.

#### 2.4.3. Statistical Analysis

The statistical difference of the coherence coefficients was assessed in each frequency bin for each word (pseudoword) using a nonparametric bootstrap procedure [68]. The bootstrap test is independent of the distribution type. For multiple comparisons, a false discovery rate (FDR) correction was calculated within each of the testing frequency bins. The statistical significance was assessed at the level of the group with the nonparametric permutation test, evaluating the coherence value with the frequency-flipped speech envelope signal. The coherence values were averaged across subjects for each frequency bin. Then, these values were contrasted, providing a different value for each sensor. In such a contrast, the sample distribution of the maximum across sensors was obtained after having randomly permuted coherence values within the subjects for a subset of 1000 permutations. The 95th percentile of this distribution yielded significance thresholds at *p* < 0.05 corrected for multiple comparisons for the initial contrast. Frequencies for which the unpermitted maximal difference exceeded the 95 percentile of this permutation distribution, were defined as frequencies of interest. The corresponding supra-threshold sensors were defined as sensors of interest for this frequency band. The coherence values were not averaged per hemisphere. They were entered separately in the analysis in case of individual introduction of the sensors, where the hemispheric information would already be coded implicitly in the sensor factor. The hemisphere factor was not entered in the model to avoid losing degrees of freedom for parameter estimation. The sensor factor was entered into the between-group comparison, while the hemisphere factor—into the within-group comparison to find hemispheric specialization for the different temporal components of speech, but not both. The random-effects by subject help to control the interindividual variability, reducing the weight of extreme observations for the group-level statistics. For standardization of data, Z-score was applied to present the average comparison, converting the data into the same scale and comparing more sets of data with different units. The averaged value (overall words or pseudowords) normalized toward the maximum value for a given stimulus and sensor. We applied this procedure for all pairs of groups: controls (Con) vs. each dyslexic subgroup and between different dyslexic subgroups. The analysis was performed with MATLAB (The Mathworks, Natick, MA, USA).

## 3. Results

### 3.1. Behavioural Data

The group with dyslexia showed a lower success rate compared to the controls in both conditions (the group with dyslexia: mean ± s.e. 63.75% ± 9.35%, words; 64.85% ± 10.45%, pseudowords; the control group: 83.1% for both conditions, *p* < 0.001; Table S2).

Some of the children with dyslexia, who performed the discrimination task with a success rate under the mean group value 64.3% in both conditions and significantly worse (word discrimination: 54.4% ± 1.22%, pseudowords: 54.4% ± 1.4%) in comparison to the controls (83.1% ± 0.73%, 83.1% ± 0.96%, *p* = 0.0001, χ^2^ > 112.9), were assigned to a subgroup with more pronounced phonological deficits (PhoDys; age range: 7.8–8.6, 7 boys, 4 girls). Other dyslexics with a success rate over their mean group value (64.3%) and with lower achievements (73.1% ± 1.21%, 73.5% ± 1.74%, *p* = 0.0001, χ^2^ > 27.2) than the control group were included in a subgroup with less pronounced phonological deficits (NoPhoDys, age range: 7.6–8.3; 9 boys, 6 girls). The reaction time for word discrimination was significantly faster for the PhoDys (1299.55 ± 17.60 ms) compared to the controls (1416.31 ± 7.06 ms, *p* < 0.01, χ^2^ = 7.65; Table S2) and the NoPhoDys (1455.63 ± 12.44 ms, *p* < 0.0001, χ^2^ = 35.03). All groups showed similar reaction times when performing the pseudoword condition (Con, 1514.27 ± 8.02 ms; NoPhoDys, 1486.01 ± 13.16 ms; PhoDys, 1482.73 ± 15.67 ms; Table S2).

In word or pseudoword stimuli, both speed and accuracy are impaired independently in both experimental conditions for dyslexia. If the pseudoword processing is particularly affected, then the (phonological) dyslexics have these particular difficulties (e.g., [69]). The paradigm manipulates the performance along two routes (lexical and sublexical). If the paradigm is well-defined to derive different types of dyslexic reading, the differences should be reflected in the brain regions at each of the conditions [18]. By a speech–brain EEG frequency analysis, we look for sensor-level specialization for the different frequency components of speech in controls and dyslexic subgroups, differentiated on a behavior level. We will define the physiological reasons for this differentiation of the groups with dyslexia. Based on neuronal oscillations, which contributed to language cognition as delta, theta, and gamma oscillations, specifically engaged by quasi-rhythmic properties of speech, we argue that they are fundamental to the differences of speech and language processing between groups. Such stimulus–brain alignment arguably results from auditory and motor tuning throughout the speech and language comprehension, allowing auditory research to contribute to the issue of how the neuronal oscillatory activity reflects linguistic and cognitive deficits in dyslexia.

### 3.2. Speech–Brain Eeg Frequency Entrainment

Brain hemispheric asymmetry was identified at the δ, β, γ–frequency entrainments relevant to the word comprehension. The planum temporale (PT) is localized posteriorly to the auditory cortex (the Heschl’s gyrus: BA41 and BA42, C5), superiorly to the superior and middle temporal gyri (MTG: middle temporal BA21, 22, 42, 41, T7; BA21, 37, 22, and TP7; [70,71]). Other areas yielding phonological processing deficit were the inferior temporal gyrus (anterior part of ITG: BA20, 38, 21, and FT9; posterior part of ITG: BA37, 19, 20, 39, and P7), and those behind the inferior frontal sulcus (IFG: Brodmann areas BA: BA45, 47, and sensor F7; BA 6, 9, 44, 45, and sensor FC5), the inferior parietal lobe (IPL; CP3: BA40—supramarginal gyrus; P3: BA39—angular gyrus). We focused on different levels of frequency entrainment in the subgroups with dyslexia when compared to controls across the frequency bands connected to the auditory processing of the speech.

#### 3.2.1. Deficiency of Delta-Frequency Entrainment in the Left Auditory Cortex of Dyslexics with More Pronounced Phonological Deficits

**Figure 1:**
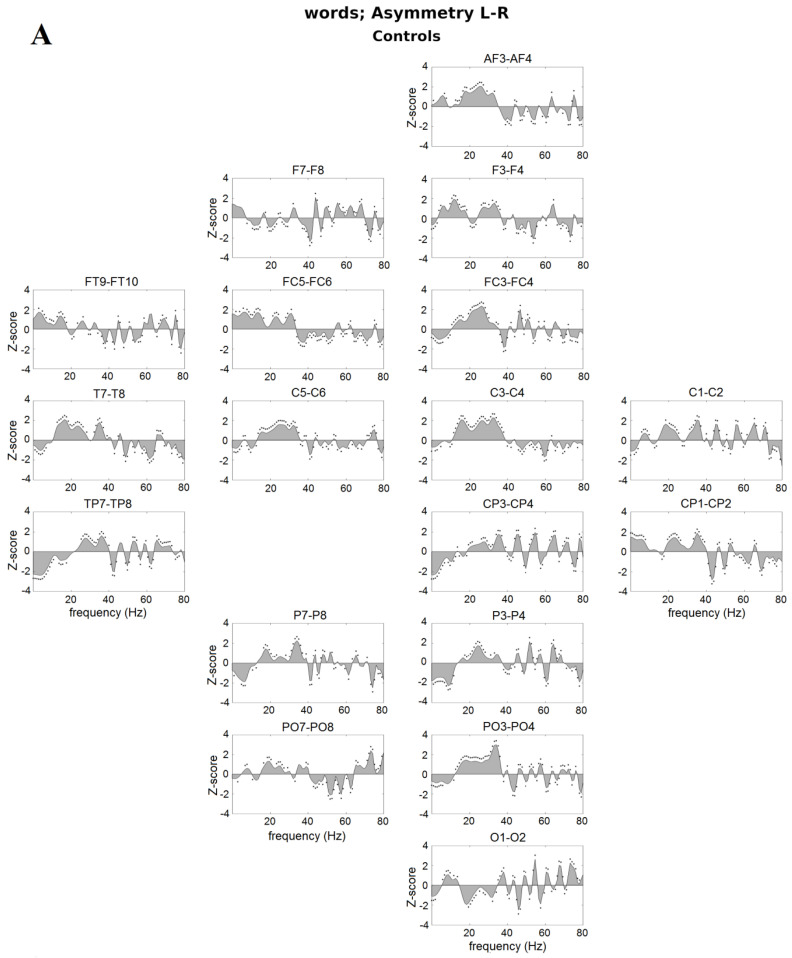

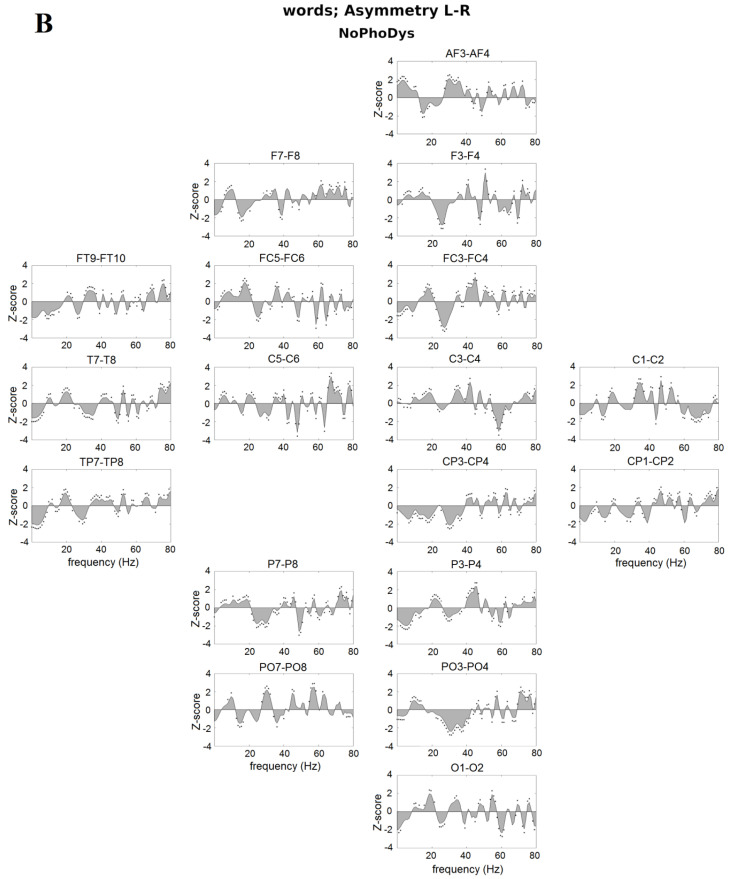

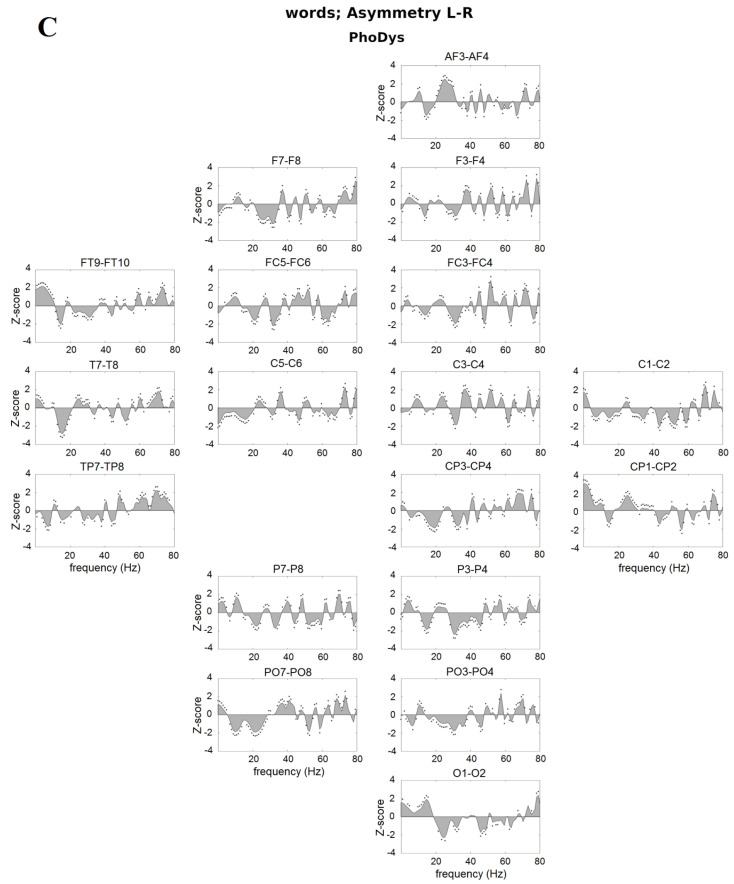
Hemispheric asymmetry of speech–brain entrainment for (**A**) controls, (**B**) NoPhoDys, and (**C**) PhoDys when word listening (left-right hemisphere). Points indicate statistically significant differences (nonparametric bootstrap, *p* < 0.05). The speech–brain entrainment on respective left hemispheric areas is represented in the upper panel of the plots and right hemispheric areas—in the lower panel of the plots.

Dominant cortical oscillatory responses as a result of acoustic modulations within a frequency range <10 Hz contain the syllable rate (2 Hz) and the prosodic rate (<4 Hz; Figure 1A–C). In the controls at 3 Hz, a significant left hemispheric dominance was observed in the IFG (FC5, *p* = 0.0341, χ^2^ = 4.48), the ITG (FT9, *p* = 0.02, χ^2^ = 5.11), and the superior parietal lobe in the 2 Hz (SPL: CP1; *p* = 8.66 × 10^−17^, χ^2^ = 69.25), and in the right MTG (TP8) and the inferior parietal lobe (IPL; CP4, P4; Figure 1A). Unlike the controls, the NoPhoDys subgroup showed strong left dominant oscillations at 4 Hz in the superior frontal gyrus (SFG, AF3) and in the right hemisphere at the MTG (TP8 and T8), IPL (P4; *p* < 1.45 × 10^−7^, χ^2^ > 27.65; Figure 1B), while the PhoDys children showed a left-hemispheric dominance in the ITG (FT9, *p* = 0.002, χ^2^ = 9.21) and SPL (CP1, *p* = 7.32 × 10^−4^, χ^2^ = 11.40, Figure 1C).

**Figure 2:**
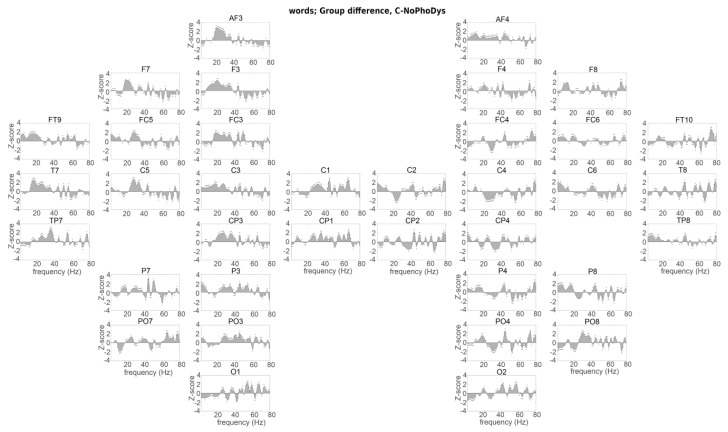
Hemispheric presentation of the group difference between controls and NoPhoDys for the word listening condition. The speech–brain entrainment on respective areas of controls is represented on the upper panel of plots and for dyslexics—on the lower panel of plots. Points indicate significant differences between the two groups (Kruskal–Wallis nonparametric test (KW test), bootstrap, *p* < 0.05).

Controls and NoPhoDys had no significant differences in more areas, especially in left PT (C5), MTG (T7). The controls showed the higher accuracy of encoding at the 4 Hz in the left hemispheric ITG (FT9), IPL (P3, *p* < 0.02, χ^2^ > 5.33), and in the right-hemispheric auditory cortex (C6, *p* = 0.01, χ^2^ = 5.80), MTG (TP8) and the inferior temporal gyrus (ITG; P8: BA37—occipitotemporal gyrus; *p* < 0.003, χ^2^ > 8.77; Figure 2). Despite the moderated δ-entrainment of NoPhoDys, they were able to report a correct number of words close to the controls. More accurate encoding of the low-frequency information corresponds to better awareness of the lexical stress from the children.

**Figure 3:**
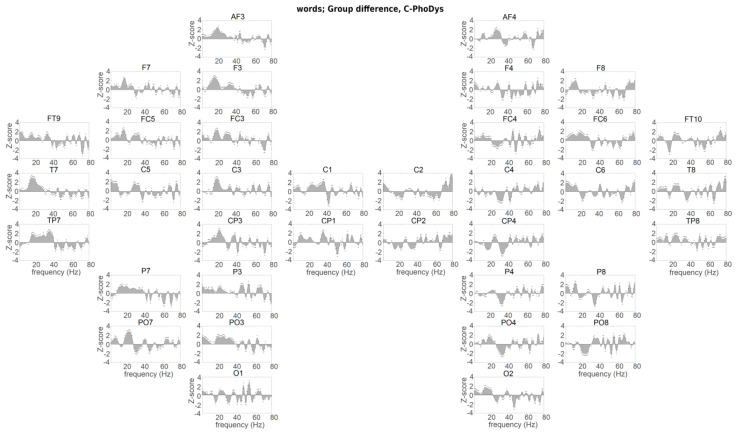
Hemispheric presentation of group difference between controls and PhoDys for the word listening condition. Same format as Figure 2.

**Figure 4:**
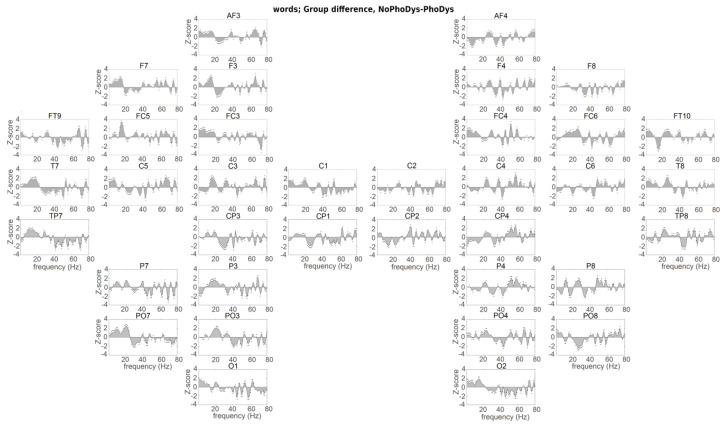
Hemispheric presentation of group difference between NoPhoDys and PhoDys for the word listening condition. The speech–brain entrainment on respective areas of NoPhoDys is represented on the upper panel of plots and for PhoDys—on the lower panel of plots. Same format as Figure 2.

The controls had significantly higher reconstruction accuracy for the syllable rate (2 Hz) than the PhoDys in the bilateral auditory cortices (C5–6, *p* < 0.03, χ^2^ > 4.57; Figure 3), and for the prosodic rate at the left ITG (FT9), right IFG (FC6), and right ITG (P8). The NoPhoDys showed maximal δ-frequency entrainment relative to the PhoDys in the left auditory cortex (C5, *p* = 1.93 × 10^−4^, χ^2^ = 13.89), the bilateral ITG (FT9–10) and the bihemispheric middle frontal gyrus (MFG; FC3–4: BA6; *p* < 0.01, χ^2^ = 6.12; Figure 4). The NoPhoDys had more correctly recognized words compared to the PhoDys.PhoDys showed the highest δ-entrainment in the bilateral IPL (P3, CP4) compared to NoPhoDys (Figure 4).

When listening to pseudowords, the controls and PhoDys showed δ-entrainment in the range under 4 Hz at the right auditory cortex (Figure S1), while the NoPhoDys—in the left auditory cortex. The controls showed better δ-entrainment in the 2–4 Hz band at the right auditory cortex, compared with the NoPhoDys, at the right inferior frontal cortex, relative to both dyslexic subgroups. The last had, however, greater δ-entrainments in the left inferior frontal and auditory cortices than normal-reading children (Figure S2). The NoPhoDys exhibited increased δ-entrainments in the left auditory and inferior frontal cortices than the other dyslexics whereas the right auditory cortex was more involved in the phonological processes in the PhoDys than in the NoPhoDys (Figure S2).

#### 3.2.2. Beta-Frequency Entrainment in the Right Auditory Cortex of Dyslexics with More Pronounced Phonological Deficits

Beta-frequency entrainments in 12–22 Hz range were dominant in the left PT (C5), MTG (T7), and the postcentral gyrus (PSTCG: C3) with a peak at 15 Hz for the controls (*p* < 7.81 × 10^−5^, χ^2^ > 15.60; Figure 1A) and with a peak at 20 Hz for the NoPhoDys in the left IFG (FC5; Figure 1B), while it was right dominant in the MTG (T8) and PT (C6) for the PhoDys (Figure 1C).

Dominant widespread β-entrainment was detected in the controls relative to the groups with dyslexia (Figure 2 and Figure 3). The between-group differences between NoPhoDys and controls showed that the NoPhoDys exhibited significantly greater β-entrainment in the right hemisphere at the PRECG (C2), PSTCG (C4), IPL (CP4), and middle occipital gyrus (MOG: PO8: BA18), but at 15 Hz in the left MOG (PO7; Figure 2). For the group differences between controls and PhoDys, the dyslexics also showed significantly greater β–entrainment more in the right hemisphere at 20 Hz in the PT (C6, *p* < 4.69 × 10^−5^, χ^2^ > 16.56), PRECG (C2), SPL (CP2), IPL (CP4), and MOG (PO8, *p* < 5.66 × 10^−4^, χ^2^ > 11.88; Figure 3). Additionally, at 16 Hz in the ITG (FT10), the left-dominant response around 15 Hz for the NoPhoDys compared to the PhoDys was present in MTG (T7, TP7), middle frontal gyrus (MFG; F3:- BA8, FC3: BA6), IPL (P3), the superior occipital gyrus (SOG; PO3: BA19), and MOG (P07), with a more expressed peak in the left PRECG (C1), bilateral IFG (F7, FC5–6), and PSTCG (C3–4; Figure 4). Beta 20 Hz peak dominated in the PhoDys in the left MFG (F3), bilateral PT (C5–6), and there was a pronounced 15 Hz peak in the right ITG (FT10, P8; Figure 4).

In the pseudoword listening, the observed β-entrainment (20 Hz) was in the right IFG of the NoPhoDys (FC6, *p* = 2.33 × 10^−8^, χ^2^ = 31.19; Figure S1). There was the same right frontal dominance at 15 Hz in PhoDys for the pseudoword listening as the listening to words but with less activity. The β-frequency entrainment was dominant at the right PT of the controls and the left PT of NoPhoDys (Figure S2). The normal-reading group showed higher β-entrainment in the right PT and IFG vs. both dyslexic subgroups. Contrary to the word condition, group-differences between PhoDys and NoPhoDys for the pseudowords showed that the NoPhoDys had a greater β–entrainment at 20 Hz than the PPhoD in left PT and the right IFG.

#### 3.2.3. Right-Hemispheric Gamma-Entrainment to the Phonemic Frequencies at 30 Hz in Dyslexics

As in normal readers, low-gamma oscillatory responses to acoustic modulations 25–35 Hz were observed in children with developmental dyslexia. In the controls, there was a γ-entrainment in left PT (C5, *p* = 1.77 × 10^−7^, χ^2^ = 27.26), MTG (T7, TP7), and ITG (P7, *p* < 4.61 × 10^−4^, χ^2^ > 12.26) in a frequency range of 25–35 Hz, which covers the phonemic sampling rate (Figure 1A). Around 40 Hz and in the upper range 55–80 Hz, the asymmetry inverted, and the responses were right-hemispheric (*p* < 0.05, Figure 1A).

Unlike controls, both dyslexic subgroups showed significant auditory entrainment to the phonemic frequencies around 30 Hz in the right PT, adjacent STS regions (C6, TP8, *p* < 1.73 × 10^−5^, χ^2^ > 14.1; Figure 1B, C). The hemispheric asymmetry in the dyslexic subgroups confirmed the reduced left-hemispheric dominance compared to the right hemisphere in this frequency domain. The right-hemispheric dominant 40 Hz was even more pronounced in the auditory cortex of NoPhoDys (C6, *p* = 1.81 × 10^−6^, χ^2^ = 18.38; Figure 1B). They additionally showed enhanced responses at frequencies above 48 Hz in the right auditory cortex. The PhoDys exhibited increased responses at 30 Hz in the right-hemispheric MTG (TP8), IPL (CP4), PSTCG (C4), MFG (FC4), IFG (FC6, F8), and ITG (FT10), unlike the left side where there was a deficit of activity (Figure 1C).

The controls relative to NoPhoDys exhibited a widespread left-hemispheric response at 30 Hz. The NoPhoDys relative to the controls had enhanced response at 55 Hz in bilateral PT (C5–6, Figure 2). The left-hemispheric 40 Hz at PT (C5) and the adjacent STS regions (T7, TP7) of PhoDys was more pronounced compared to the controls, and in the ITG (P7), IPL (CP3), peaking at the PRECG (C1; Figure 3). The PhoDys showed more pronounced entrainment around 40 Hz in both hemispheres relative to the NoPhoDys (Figure 4). These results, therefore, not only indicated the atypical sensitivity of the left auditory cortex to sound modulations in the 25–35 Hz frequency range but also increased bilateral sensitivity to the faster modulations in children with dyslexia than in controls. The phoneme rate, in the groups with dyslexia, can be shifted either up or down. The shifting up can lead to a deficit in phonological/verbal working memory. The altered asymmetry in the 25–35 Hz range in dyslexia was accompanied by increased entrainment in the auditory cortices at high frequencies (above 48 Hz), suggesting a hearing “reassembly”. Hence, these “abnormal” high-frequency oscillations in the left auditory cortex of the groups with dyslexia could explain the poor phonological working memory. The NoPhoDys had a stronger resonance than controls in the left auditory cortex (C5, Figure 2) at a wide range of frequencies (45–65 Hz), whereas PhoDys relative to the controls—in the adjacent STS regions (T7, TP7) and ITG (P7, Figure 3). Only the NoPhoDys exhibited stronger resonance than PhoDys at the frequencies (45–65 Hz) in the bilateral auditory cortices (C5–6), while PhoDys—in left MTG (TP7) and ITG (P7; Figure 4). The atypical γ–entrainment for the NoPhoDys was not strictly limited to the auditory cortex and extended to the right-hemispheric MFG (FC4), PSTCG (C4), SPL (CP2), IPL (CP4, P4), and ITL (P8). Whereas, this γ–entrainment, in PhoDys, was spread in the left hemisphere from the adjacent STS regions to the PRECG (C1), PSTCG (CP1), superior occipital gyrus (SOG; PO3-BA19), and bihemispheric MOG (P07, O1–2; Figure 4).

Hemispheric asymmetry, when listening to pseudowords, was the opposite of the one shown in the word condition (Figure S1). In the right hemisphere the 25 Hz was dominant in PT and IFC for controls compared with the left hemisphere (C6, FC6, *p* < 1.5166 × 10^−4^, χ^2^ > 14.3515; Figure S1). Both dyslexic groups had a dominant gamma frequency in the 35–45 Hz range at the left PT and IFC, peaking at 65 Hz in the right IFC (Figure S1). NoPhoDys additionally showed enhanced responses at frequencies above 48 Hz in the left auditory cortex (Figure S1). Group differences in listening to pseudowords showed that PhoDys and NoPhoDys had a left-hemispheric 40 Hz in PT and IFG compared to the controls, more pronounced in PhoDys at left IFG (Figure S2). The PhoDys had a more pronounced response at 46–65 Hz in the right hemisphere compared to NoPhoDys (Figure S2).

The average EEG γ-frequency entrainment around 40 Hz was generally higher in the right than in the left auditory cortex when listening to words and in the left auditory cortex when listening to pseudowords.

#### 3.2.4. Strength of the Multi-Frequency Entrainment in Concordance with Psychometric Indicators

The right orbitofrontal gyrus, left MTG, SPL were involved in low-level δ-entrainment for both dyslexic subgroups than controls. In the left hemisphere, NoPhoDys had atypical higher speech–brain δ-entrainment than controls at the dorsolateral prefrontal (DLFC), premotor cortex (PMC), supplementary motor area (SMA), and in the bilateral intermediate frontal gyri (including frontal eye fields, MFG) however, peaking in the right occipital areas, (V2 and V3 in MOG). In the left hemisphere, moderate δ-response for PhoDys, with regard to the normal-reading children, was observed in the IPL (angular gyrus), occipitotemporal area (including fusiform gyrus), and MOG. A strong speech–brain δ-activity in NoPhoDys (vs. PhoDys) covered the left hemispheric auditory cortex, DLFC, anterior temporal lobe (ATL), and the bilateral MFG, PMC, SMA, and MOG. Otherwise, PhoDys showed a stronger δ-response in the left occipitotemporal area and S1, peaking in the left IPL (angular gyrus) and the right IPL (supramarginal gyrus).

The subgroups with dyslexia showed higher beta-responses (vs. the controls) at the right-hemispheric primary somatosensory cortex (S1) and the associative visual cortex. Furthermore, compared with controls, the right motor cortices (PMC, M1, and SMA) and the supramarginal gyrus were more involved in the β-entrainment for NoPhoDys, and for PhoDys, in the right PT and SPL with a maximum in the right ATL. Similarly, as the controls, the left-hemispheric lower β-frequency response (15 Hz) for the NoPhoDys (with regards to PhoDys) was present in the temporal, frontal, superior parietal, inferior parietal lobes, associative visual cortex, bilateral DLFC, motor cortices, and S1, with more pronounced peaks in the IFC (pars triangularis) and the occipitotemporal area. Higher β-frequency range (20 Hz) in PhoDys dominated in the left MFG, PMC, SMA, bilateral PT, and in the right ATL and the associative visual cortex.

The left dominant 30 Hz γ-entrainment, typical for the normal-reading children to phonemic frequency, was more pronounced in the right PT and adjacent STS regions in both dyslexic subgroups. Additionally, the left hemispheric γ-entrainment around 40 Hz prevailed in the PhoDys than in the other groups, shifting up to higher-frequency γ-entrainment (45–65 Hz) on the left adjacent STS regions and ITG of PhoDys and in the auditory cortices of NoPhoDys (>55 Hz) as regards the other groups. Even this atypical higher γ-entrainment in NoPhoDys was spread over the right hemisphere at the PMC, SMA, S1, somatosensory association cortex, IPL, and occipitotemporal area. The higher γ-frequency overactivity for PhoDys (vs the other groups) also covered the left hemispheric PMC, M1, SMA, S1, somatosensory association cortex, and occipital cortices.

The statistical comparisons (one-way ANOVA) of the psychometric results between both subgroups with dyslexia showed statistically significant differences, where the achievement of children with the pronounced phonological deficit was significantly lower in the writing of words, pseudowords, sentences, and the subtest reading with comprehension of the homonym meaning in the DDE2 test battery (Table 1). The comparison of the results in the “reading abilities” battery of the subgroups with dyslexia showed that the PhoDys produced significantly less correct answers and slower performance in both phonological tasks. Additionally, this subgroup showed the worst time in the text reading than the group with the less phonological deficit (Table 1). The dictations in both test batteries were most difficult for children with a severe phonological deficit.

Bulgarian children with dyslexia are hampered in various phonological and writing skills, and the orthographic processing. However, some children with dyslexia, as those identified with the more pronounced phonological deficits, are more severely impaired. Children with PhoDys tend to exhibit more difficulties in reading and phonological processing than other dyslexic readers have.

In speech processing, PhoDys relies more on the ventral stream, involved more superior and middle portions of the temporal lobe in the boundary of the dorsal pathway (the temporal and parietal lobes). However, NoPhoDys rely on the auditory cortex, posterior frontal lobe, and articulatory brain representations. The neuropsychological evidence for the dyslexic groups supposes that partially distinct neural substrates as a dorsal and a ventral route, respectively, underlie sublexical and lexical processes. For PhoDys, the lexicosemantic route involved the left temporal language area and the posterior part of the middle temporal gyrus. While for NoPhoDys, the sublexical route involved the left-lateralized superior temporal area and the inferior frontal gyrus. Generally, as in the visual modality, due to anterior and middle temporal lobe deficits, affecting primary ventral, occipitotemporal, and lexical route, PhoDys relies more on ventral (occipitotemporal lexical) brain regions, whereas the NoPhoDys uses more of the dorsal (occipitoparietal sublexical) route. When the lexical route has deficits, as in the case of NoPhoDys, partial compensation appears to be possible by over-recruitment of the slower, attention dependent, sublexical one. The phonological failure in PhonDys occurred due to impairment in the dorsal auditory pathway [21,72,73]. It reflected the writing disability of words, nonwords, and sentences under dictation (1.3, 1.4, and 1.5 in DDE-2; 2.4 in “Reading abilities”), and reading failure (1.6 in DDE-2; 2.1–2.3 in “Reading abilities” [27]). However, this is due to a deficit in the auditory-related areas in the superior temporal lobe, but not motor areas (phonological dyslexia). This group is unable to exhibit the multisensory integration with the visual speech information, which is also a function of cross-sensory integration but is not a motor speech-mediated function [74]. NoPhoDys has no deficit in the auditory-related areas as visual dyslexia. However, these dyslexics cannot map the sensory or phonological representations to the lexical conceptual presentations due to a more expressed disability of the ventral pathway [27].

## 4. Discussion

This study revealed that when listening to words, the low-frequency speech–brain entrainment is significantly worse in children with phonological dyslexia and with visual dyslexia, compared to control children, and could be differentiated by both subgroups. The data showed significantly less accurate brain encoding and understanding of the low-frequency speech information by dyslexic participants compared to the typically developing children of the same age. These significant group differences in the speech–brain encoding accuracy remained strong when data analysis is limited to words that were correctly recognized by the participants, excluding differential stimulus engagement. Furthermore, this low-frequency speech–brain encoding accuracy is strongly associated with the individual differences in phonological awareness. These results have essential consequences for the cause of dyslexia and the decrease of “the phonological deficit” in the affected children.

The neural encoding of the speech information was atypical in the EEG delta frequency-band suggested language disabilities at the level of speech prosody and syllabic analysis. The amplitude modulation structure of early childhood speech shows a modulation peak at 2 Hz for the “prosodic rate” [75], not the modulation peak at 4–6 Hz, found in adults [76]. These different modulation peaks suggest that, at the beginning of the development, a precise encoding of the low-frequency speech information would play a crucial role in the creation of a phonological dictionary [74]. Children who are relatively insensitive to the low-frequency speech information would benefit less from the prosodic information of the early childhood speech as they formulate their lexical neural representations [75]. In the word-listening task, we found that the children with phonological dyslexia showed reduced delta-oscillatory entrainment originating from the right primary auditory cortex (Figure 1 [55]). Parts of speech that are in the delta frequency range are difficult to encode. The encoding does not improve with the age of development [55]. In dyslexia, the inverse functional relationship between the right hemisphere neural oscillations and those in the left inferior frontal brain areas, involved in the comprehension of speech, is perturbed mainly in dyslexic children with less pronounced phonological deficits [55]. The neural alterations showed disturbed neural encoding in both syllable and prosodic linguistic levels in dyslexic children. Delta-frequency speech–brain entrainment is presented in several anatomical areas, including Heschl’ gyrus, planum temporale, superior temporal gyrus, inferior parietal lobe, and inferior frontal gyrus. It suggests that the group differences appear relatively early in neural processing during auditory encoding. The present study used natural speech stimuli, not noise-vocally synthesized speech [48], which in other studies reduced the neural involvement of higher linguistic areas such as the angular gyrus [48] and increased the engagement of lower-level areas like the adjacent STS regions, the primary auditory cortex [77]. The brain areas whose delta frequency oscillations are synchronized with the right and left auditory cortex, right and left STG, right middle temporal gyrus, left IFG, and left anterior temporal region, spread to the neural feedback network with the origin in the auditory cortex [55]. Although IFG’s contribution cannot be ruled out, it is assumed that the primary auditory cortex and adjacent STS region are probably the most relevant areas to the encoding precision. Both dyslexic subgroups, concerning the normal readers, had a deficit of δ-entrainment in the left ATL and IFG, the right auditory cortex, and inferior temporal gyrus. It was revealed additionally in the right MTG for non-phonological dyslexics, whereas for phonological dyslexics, in the left auditory cortex. These areas are involved in more general semantic integration due to not having a specific role in the mapping between phonological and conceptual representations [27]. A weak δ-entrainment for both subgroups with dyslexia, compared to the normal-reading children, was observed at the right orbitofrontal cortex, the left MTG, and the left SPL. At the IPL (angular gyrus) and IFG, the controls exhibited greater δ-entrainment than both dyslexic subgroups. The children with phonological dyslexia showed greater δ-entrainment in IPL (angular gyrus) comparing with the other dyslexics. Similarly, as the controls, the non-phonological dyslexics, compared with the other dyslexics, showed stronger δ-entrainment in the left auditory cortex, IFG, DLFC, and ATL, and the bilateral MFG, PMC, and SMA, and occipital cortices. The deficit in frontal or inferior parietal areas in the left hemisphere can cause deficits in discrimination of speech syllables, included generalized attentional deficits and phonological working memory deficits [73]. Due to the strong connection between the inferior parietal lobe and the inferior frontal gyrus, the dysfunction of one of them could have an impact on the mode of operation in the other area. Therefore, the dyslexic subgroups had different patterns of brain activation, indicating strategies of differential phonological processing as a basis for meaningful interpretation. The data for the δ–speech envelope encoding showed that the prosodic and syllable information (both are in the delta range of the stimuli) were not coded exactly when speech listening. Atypical neural δ-entrainment to amplitude modulations of speech, slower than 10 Hz, may contribute to the phonological processing disturbances found in developmental dyslexia.

The study of hemispheric asymmetry showed a widespread higher β-entrainment in typically developing children and children with visual dyslexia than the group with phonological dyslexia. The subgroups with dyslexia, in comparison with the controls, exhibit right-hemispheric greater β-responses at the primary somatosensory and associative visual cortices and in the supramarginal gyrus and motor cortices—for non-phonological dyslexics, and in the PT and SPG, peaking at the ATL—for the phonological subtype. A higher beta-frequency entrainment was also found in the frontal areas of the typically developing children than those in NoPhoDys. The use of natural speech as a stimulus has placed higher requirements during language processing related to cognitive skills such as working memory. The controls had stronger beta-frequency entrainment compared to the dyslexic subgroups, suggesting that differential beta activity for children with dyslexia does not contribute enough to the encoding of speech, especially in those with severe phonological deficits. The left-dominant low-frequency β-response in the non-phonological dyslexics than those with severe phonological deficits was present in the temporal and frontal lobes, superior and inferior parietal lobe, motor cortices, somatosensory cortex, the occipitotemporal area, and peaking in the IFG. High-frequency β-entrainment in the phonological dyslexics dominated at the left intermediate frontal, premotor, SMA, the bilateral auditory cortices, and the associative visual cortex, with a maximum in the right ATL. The insufficient low-frequency beta changes over the inferior frontal and parietal areas of dyslexics with severe phonological deficits, compared to those with less dominant phonological deficits, correspond to their worse phonological short-term memory. The study has shown that the beta-frequency activity of the left-hemispheric supplementary motor areas, closely linked to the phonological rehearsal mechanism, also contributes to the speech comprehension in the phonological dyslexics than in the non-phonological ones when listened to words and pseudowords [2,78]. The enhanced 20 Hz entrainment at the phonological dyslexics could be associated with a compensatory mechanism for the inefficient phonemic-rate information processing, corresponding to the poorer phonological skills. The beta-entrainment at 15 Hz for non-phonological dyslexics found in the temporal lobe, similarly to those in the normal-reading children, could be associated with the semantic prediction and comprehension of the word meaning. It was insufficiently in the phonological dyslexics.

The results reported here are consistent with the neural explanation for speech encoding [56] and the temporal sampling theory in developmental dyslexia [49]. According to that, the speech is encoded in part by neural oscillations in the delta, beta, and gamma frequency ranges in the auditory cortex, corresponding to the amplitude modulations of the speech signal [44,79]. We confirmed that the auditory gamma-frequency entrainment within a limited 25–35 Hz acoustic modulations in the fluent reading children was stronger or selectively amplified rather in the left than in the right auditory cortex that improves the phonemic sampling ability of the left auditory cortex [80]. We observed maximum low-gamma frequency oscillations in both the PT and STS region for the typical readers, but left-dominant ones were less pronounced in the adjacent STS regions. PT and STS regions represent two consecutive steps in speech processing. The adjacent STS region combines auditory and visual speech events because of its high position in the auditory hierarchy. Unlike controls, the children with dyslexia do not exhibit the hallmarks of lateralization of low gamma-frequency entrainment. The frequency entrainment, at 25–35 Hz, was reduced in the left auditory cortex with a maximum deficit of 30 Hz. For phonemic signs, this deficit is manifested as an impairment of the selective extraction and encoding from the left hemisphere, and thus disturbed the interhemispheric sorting of auditory information. The left-hemispheric dominance within the 25–35 Hz range, corresponded to the phonemic rate for the controls, cannot be found in the children with dyslexia. It supports the hypothesis that left hemisphere dominance in low-gamma frequency activity is the basis of good phonological abilities and contributes to reading skills. Behavioral measures and left-hemispheric dominance of the EEG oscillations at exactly 30 Hz, where is observed the maximum for the controls, reveal opposite effects for the subgroups with dyslexia. Given that there is no difference in the preferred hand between the groups, the subgroups with dyslexia compensate with the right auditory cortex for insufficient phoneme analysis in the left one. The right auditory cortex of the children with dyslexia showed increased entrainment of 30 Hz modulations versus the controls, and compared to their left auditory cortex. Compensation of the left-hemispheric deficit from the right hemisphere is such an adaptation that rarely gives full behavioral improvement. The enhanced gamma-entrainment in the right auditory cortex may have a positive effect on the phonological analysis. However, it may not benefit the phonological output processing. It reflects the significant negative effect close to 40 Hz at the left ATL, middle temporal, precentral, postcentral, superior parietal, inferior parietal, and occipital cortices of children with predominant phonological deficits than the other subgroup with dyslexia. It is similar to the higher gamma effect at the left IFG of the children with less dominant phonological deficits. Insufficient phonological perception can be compensated by the right auditory cortex, whereas the phonological production cannot, probably because it relies on an expanded highly lateralized neural network covering the inferior frontal and parietal cortex [47]. The low gamma-frequency peculiarity may encompass the speech perception/production circuit due to widespread abnormalities in the articulator areas (motor and somatosensory cortices) of the children with predominant phonological deficits than in the other groups [81]. The dyslexics with less dominant phonological deficits, compared to the controls, exhibited over-normal entrainment of bilateral PT to rapid temporal modulations in the 55–70 Hz range, suggesting that their auditory cortices overamplify the acoustic speech stimuli. The abnormal presence of high-frequency auditory oscillations in dyslexia may indirectly affect phonological/verbal memory. It would require a higher amount of subphoneme perceptual pieces per temporal bin integrated into the delta-frequency processes [53]. The left-hemispheric dominant oversampling in the middle temporal and the angular gyri for the phonological dyslexics, compared to the non-phonologic dyslexics, may be associated with poor phonological working memory. The storage of auditory information in short-term memory is equivalent to filling a limited capacitive buffer with consecutive representations. Hence, the system encodes the stimulus with an abnormal temporal symbolic representation with rare or fast-moving codes [82,83]. Both the left inferior frontal cortex and the adjacent STS region cannot align their oscillatory properties with those of the auditory cortex in a high-frequency gamma-band. It may indicate their difficult collaboration for performing general functions, such as verbal memory [47].

This approach suggests that the deficits on different linguistic levels can be addressed to semantic, lexical, and phonological processing. The results revealed deficits in two processing pathways in developmental dyslexia. It supports the evidence that these pathways can be selectively impaired. The translation of sound into motor speech is performed without linking to the conceptual system. The inability to read because of temporary deactivation of the motor speech system does not preclude the ability to comprehend spoken language [17,35]. The right frontoparietal homologous areas might be involved in compensation processes in developmental dyslexia that were observed in dyslexics but not in normal readers [35]. The neuronal mechanisms, specialized for detecting auditory stimulus timing and change, were dysfunctional in children with phonological dyslexia [3,72]. The auditory dissociation in the performance of dyslexics suggests a submodality division similar to those in the visual system [3,84]. The auditory temporal processing in coding information discriminated phonemes in the speech was disturbed in children with a severe phonological deficit.

## 5. Conclusions

The present study is evidence that different patterns of cortical reorganization based on the left or right hemisphere lead to differential profiles of dyslexia. That contributes to defining the neurological outcome in dyslexia. Therefore, the differential activation profiles between the children with dyslexia and normolexics can be found, depending on the cognitive deficit that underlies the difficulty of reading in children.

## Figures and Tables

**Table 1:** The group data of the test battery DDE-2 and test battery “Reading abilities” in standard scores.

Test Battery	Controls	Less Phon.	Severe Phon.	Severe Phon. vs. Less Phon.
	Mean ± s.d.	Mean ± s.d.	Mean ± s.d.	*p*
1. DDE-2				
1.1. Word reading				
Accuracy	106 ± 5.58	93.0 ± 13.29	78.5 ± 19.10	0.077
Speed	132 ± 0.76	90.6 ± 11.12	79.9 ± 11.68	0.604
1.2. Nonword reading				
Accuracy	102 ± 4.65	87.9 ± 15.78	80.9 ± 19.57	0.167
Speed	118 ± 0.68	92.9 ± 8.34	88.3 ± 10.99	0.206
1.3. Word writing				
Accuracy	115 ± 6.49	97.1 ± 10.36	73.6 ± 11.77	0.0001 ***
1.4. Nonword writing				
Accuracy	104 ± 4.25	105.0 ± 9.31	78.4 ± 12.69	0.000 ***
1.5. Dictation				
Accuracy	112 ± 4.82	101.7 ± 9.75	75.1 ± 9.79	0.0001 ***
1.6. Homonyms				
Accuracy	112 ± 1.82	103.1 ± 10.54	91.2 ± 18.92	0.041 *
1.7. Search for misspellings of words				
Accuracy	112 ± 4.45	110.5± 11.56	107.0 ± 17.65	0.526
2. Test battery “Reading abilities”				
2.1. Phonological task ”without a first sound-letter”				
Correct answers	9.20 ± 1.87	6.86 ± 1.30	4.17 ± 2.78	0.002 **
Execution time	34.60 ± 10.50	43.00 ± 13.92	82.06 ± 45.03	0.003 **
2.2. Phonological task ”without a last syllable”				
Correct answers	8.05 ± 2.08	7.13 ± 2.06	5.17 ± 2.69	0.030 *
Execution time	37.50 ± 8.80	41.80 ± 10.28	87.88 ± 50.74	0.002 **
2.3. Text reading				
Correct answers	129.41 ± 3.43	118.00 ± 9.84	121.29 ± 5.49	0.244
Execution time	104.77 ± 29.00	122.73 ± 37.23	252.29 ± 183.74	0.012 *
2.4. Dictation filling in a missing compound word				
Correct sentences	21.00 ± 5.85	14.46 ± 3.79	7.41 ± 5.22	0.0001 ***

*** *p* < 0.001, ** *p* < 0.01, * *p* < 0.05.

## Supplementary Materials

The following are available online at https://www.mdpi.com/2076-3425/10/12/920/s1, Supplementary Files 1: Consent form and Information list. File 2: Participant information sheet. File 3: with words pseudowords list. Figure S1: Hemispheric asymmetry (left–right hemisphere) for the pseudoword listening condition. Controls are in the first column, NoPhoDys in the second and the PhoDys in the third. Points indicate statistically significant differences (KW test, bootstrap, *p* < 0.05). Same format as Figure 1. Figure S2: Group difference for the pseudoword listening condition between controls and NoPhoDys, controls and PhoDys, NoPhoDys, and PhoDys. The left column is left IFG and PT; their respective right hemispheric areas are represented in the right column. Points indicate significant differences (KW test, bootstrap, *p* < 0.05). Same format as Figure 2. Table S1: The group data presented in standard scores. Table S2: The behaviour parameters of the groups are represented by the percent of the correct answers and reaction time.
