# Supplementary material for: Speech–Brain Frequency Entrainment of Dyslexia with and without Phonological Deficits
Source: Brain Sci. 2020 Nov 28;10(12):920. doi: 10.3390/brainsci10120920 (PMC7760068; doi:10.3390/brainsci10120920)
Supplement: Supplementary file 1 [file brainsci-10-00920-s001.zip › supp files/Supp File 1 Consent form and Information list BrainSci-981931_original.docx]

ИНФОРМАЦИОНЕН ЛИСТ ЗА УЧАСТНИКА В ИЗСЛЕДВАНЕТО

Заглавие на проекта:

| Комплексен интегриран мулти-компонентен проект при изследване на дислексия на развитието |
| --- |

По договор ДН05/14/2016 г. с Фонд Научни изследвания

Ръководител: доц. Юлиана Душанова, тел. 879137432

ОБЯСНЕНИЯ И ИНФОРМАЦИЯ ЗА УЧАСТНИКА В ИЗСЛЕДВАНЕТО ОТНОСНО СЪЩНОСТТА НА ПРОЕКТА:

Уважаеми Родители,

Бихме искали да Ви помолим за участието на Вашето дете в изследователски проект с горепосоченото заглавие ”Комплексен интегриран мулти-компонентен проект при изследване на дислексия на развитието” в по-долу описаните неврофизиологични изследвания и в набор от обучителни зрителни и паметови задачи *(последното липсва при здрави доброволци)*.

Участието е изцяло доброволно и ако не желаете, не трябва да се включвате в това изследване.

Проектът се провежда от (мултидисциплинарен) екип, включващ изследователи от Институт по Невробиология – БАН, Институт за изследване на населението и човека – БАН, Логопедичен център към Министерство на образованието и науката – гр. София

Участието на Вашето дете ще бъде в планирано проучване с цел задълбочено изследване на неврофизиологични характеристики на деца с обучителни затруднения чрез неинвазивна ЕЕГ образна диагностика, безопасна за участниците. С нея ще се изследва активирането на специфични мозъчни области с набор от слухови, зрителни, речеви и паметови задачи. Изучаване на механизмите на дислексия на развитието и промените след тренировка със зрителни задачи изяснява причините за това нарушение в детска възраст. Установяването на определени електрофизиологични показатели, както и придобиването на знания, ще даде възможност да се развият неинвазивни и немедикаментозни терапии, насочени към подобряване на функциите, подпомагащи обучителните способности в детска възраст. Някои от тези проучвания не са част от рутинната оценка на деца с обучителни проблеми. Участието на Вашето дете в това изследване не означава, че има някакъв тип заболяване, тъй като е насочено към здрави лица.

В хода на проекта, данните от проведените изследвания и получените резултати ще бъдат използвани единствено за научни цели, в това число и научни публикации, в които няма да се изписват имена.

Не очакваме тези изследвания да причинят каквито и да било физиологични или психични безпокойства или вреди. Провеждането на тези изследвания не носи допълнителен риск за Вашето дете и неговото здраве.

Никаква информация, получена по време на изпълнението на изследователския проект, която засяга лично Вас и Вашето дете, няма да бъде свободно разпространявана или споделяна с трети лица, освен в случаите, изрично указани в ЗЗЛД.

БАН не осигурява застраховка на лицата, участващи в изследването.

В хода на проекта **няма да бъдат провеждани генетични изследвания.**

При започването му, лекар-член на изследователския екип, ще Ви зададе въпроси относно някои лични данни, заболявания на вашето дете, от които страда сега или е страдало в миналото, както и за приемани от него медикаменти. Получената информация ще бъде кодирана и няма да се разкрива пред трети страни.

При осъществяване на проекта **не се налага да бъдат провеждани изследвания и процедури, освен обичайните**, задължително изисквани при изследвания на деца с обучителни проблеми.

**Първа сесия:**

Невропсихологичен тест (въпроси, тестове за паметта, език), често използван за откриване на проблеми с когнитивните функции и психологичен тест за оценка на дислексия на развитието. Всеки тест е с продължителност 60 минути и ще се провежда от психолог (лекар) и логопед.

**Втора сесия:**

След психологичния тест в друг ден, електрофизиологично изследване ще бъде проведено от изследовател към Института по Невробиология (следобед или сутрин). Електроенцефалографското изследване (ЕЕГ) е част от рутинна оценка на пациенти със зрителни, слухови и паметови проблеми. При тази процедура, детето ще седи на стол с поставена на главата ЕЕГ шапка със сухи електроди; няма да се ползват електродни гелове и се гарантира пълна безопасност на детето. Тази сесия ще се извърши на два етапа: Първият ще е с продължителност 30 минути, а вторият - на следващия ден за около 20 минути. Ако детето усети дискомфорт, по всяко време можете Вие или Вашето дете да прекъсне/те изследването.

**Трета сесия:**

**Тренировка със зрителни и паметови задачи**

Провежда се след завършване на ЕЕГ изследването и следва експериментален протокол, съставен и воден от психолог към Института за изследване на населението и човека. Провежда се в период от 3-4 месеца в единични кратки сесии по 15-20 мин два пъти седмично.

**Четвърта сесия:**

След тренировачния период ще има единична ЕЕГ сесия (30 минути) с речева, слухова и зрителна задача. За повече информация можете да се върнете в предишния раздел, в който се обяснена ЕЕГ процедурата. Присъствието на родител се допуска при провеждане на ЕЕГ сесиите.

Ако решите да участвате, цялата информация за Вас и Вашето дете ще остане поверителна.

Ако по което и да е време имате коментари по отношение на тези изследвания или въпроси относно Вашите права и тези на вашето дете като изследвано лице, трябва да се свържете със съответната служба на Института по невробиология, БАН.

Като се подписвате по-долу, Вие потвърждавате, че сте прочели и разбрали тази форма и доброволно желаете Вашето дете да участва в изследването.

**ФОРМУЛЯР ЗА ИНФОРМИРАНО СЪГЛАСИЕ**

Заглавие на проекта:

| Комплексен интегриран мулти-компонентен проект при изследване на дислексия на развитието |
| --- |

Моля, подчертайте **Да** или **Не** за всички посочени по-долу твърдения (**подчертава се вярното твърдение**).

## Бях помолен да се съглася сам Да Не

Прочетох Информационния лист на участника в изследването Да Не

Дадена ми бе възможност да задам всички важни за мен и

моето дете въпроси и да обсъдя този проект Да Не

Получих удовлетворяващи ме отговори

на всички мои въпроси Да Не

Получих достатъчна информация относно проекта Да Не

Удостоверявам с подписа си, че бях устно и детайлно предварително информиран/а, зададох въпросите си и получих отговори на тях от………………………………………………….......................................................................

(име на изследователя)

Информиран/а съм, че провежданите изследвания са в съгласие с декларацията от Хелзинки (2008), относно етичните принципи на медицинските изследвания при хора и са одобрени от Комисията по био етика при Институт по невробиология, БАН.

Ако резултатите от това изследване бъдат публикувани, то самоличността на изследваното лице ще остане неразкрита.

Съгласен съм моето дете да участва доброволно в изследването.

Разбирам, че съм свободен/свободна да се откажа от участие на моето детето в изследването по всяко време, без да давам обяснения за отказа си и без това да повлияе на полагащите се в бъдеще медицински грижи.

Име, презиме, фамилия на изследваното дете:

……………………………………………………………………...............................................

Име, презиме, фамилия на родител:

……………………………………………………………………...............................................

Подпис на родител:……………………..

Изследователят потвърждава чрез подписа си, че е предоставил на изследваното лице необходимата информация за настоящето изследване.

……………………………

(Подпис на изследователя)

Дата:……………….
