# Supplementary material for: Speech–Brain Frequency Entrainment of Dyslexia with and without Phonological Deficits
Source: Brain Sci. 2020 Nov 28;10(12):920. doi: 10.3390/brainsci10120920 (PMC7760068; doi:10.3390/brainsci10120920)
Supplement: Supplementary file 1 [file brainsci-10-00920-s001.zip › supp files/Supp File 2 PARTICIPANT INFORMATION SHEET BrainSci-981931_translation.docx]

**INFORMATION SHEET FOR PARTICIPANT IN THE STUDY**

**Project title:**

Complex integrated multi-component project in the study of developmental dyslexia

Under contract DN05 / 14/2016 with the Scientific Research Fund

Supervisor: Assoc. Prof. Juliana Dushanova, tel. 879137432

EXPLANATIONS AND INFORMATION FOR THE PARTICIPANT IN THE STUDYABOUT THE NATURE OF THE PROJECT:

Dear Parents,

We would like to ask you for your child's participation in a research project with the above title "Complex integrated multi-component project in the study of developmental dyslexia" in the neurophysiological studies described below and in a set of educational visual and memory tasks (*the latter is missing in healthy volunteers*).

Participation is entirely voluntary and you should not participate in this survey if you do not wish to do.

The project is implemented by a (multidisciplinary) team, including researchers from the Institute of Neurobiology - BAS, Institute for Population and Human Studies - BAS, State Logopedic Center,r at the Ministry of Education and Science – Sofia.

Your child's participation will be in a planned study to thoroughly examine the neurophysiological characteristics of children with learning disabilities through non-invasive EEG imaging that is safe for participants. It will examine the activation of specific brain areas with a range of auditory, visual, speech, and memory tasks. Studying the mechanisms of developmental dyslexia and changes after training with visual tasks clarify the causes of this disorder in childhood. The establishment of certain electrophysiological indicators, as well as the acquisition of knowledge, will enable the development of non-invasive and non-drug therapies aimed at improving the functions supporting the learning abilities in childhood. Some of these studies are not part of the routine assessment of children with learning difficulties. Your child's participation in this test does not mean that there is any type of disease, as it is aimed at healthy people.

In the course of the project, the data from the conducted research and the obtained results will be used only for scientific purposes, including scientific publications in which names will not be written.

We do not expect these studies to cause any physiological or mental distress or harm. Conducting these tests does not pose an additional risk to your child and his health.

No information obtained during the implementation of the research project that concerns you personally and your child will be freely disseminated or shared with third parties, except in the cases explicitly stated in the PDPL (personal data protection law).

BAS does not provide insurance to the persons participating in the study.

**No genetic research will be conducted** during the project.

At the beginning, a doctor who is a member of the research team will ask you questions about some personal data, your child's illnesses that he or she is suffering from now or has suffered from in the past, as well as medications he or she is taking. The information received will be encrypted and will not be disclosed to third parties.

During the implementation of the project **it is not necessary to conduct research and procedures, except for the usual ones,** which are obligatorily required for research of children with learning problems.

**First session:**

Neuropsychological test (questions, tests for memory, language), often used to detect problems with cognitive functions and psychological test to assess developmental dyslexia. Each test lasts 60 minutes and will be conducted by a psychologist (doctor) and a speech therapist.

**Second session:**

After the psychological test on another day, an electrophysiological examination will be performed by a researcher at the Institute of Neurobiology, BAS (afternoon or morning). Electroencephalographic examination (EEG) is part of a routine assessment of patients with visual, auditory and memory problems. In this procedure, the child will sit in a chair with an EEG cap with dry electrodes on his/her head; electrode gels will not be used and complete safety of the child is guaranteed. This session will take place in two stages: The first will last 30 minutes, and the second - the next day for about 20 minutes. If the child feels uncomfortable, you or your child can stop the test at any time.

**Third session:**

**Training with visual and memory tasks**

It is performed after the completion of the EEG examination and follows an experimental protocol, compiled and maintained by a psychologist at the Institute for Population and Human Studies, BAS. It is held for a period of 3-4 months in single short sessions of 15-20 minutes twice a week.

**Fourth session:**

After the training period there will be a single EEG session (30 minutes) with speech, hearing and visual task. For more information you can return to the previous section, which explains the EEG procedure. The presence of a parent is allowed during the EEG sessions.

If you choose to participate, all information about you and your child will remain confidential.

If at any time you have comments regarding these studies or questions about your rights and those of your child as a subject, you should contact the relevant service of the Institute of Neurobiology, BAS.

By signing below, you confirm that you have read and understood this form and voluntarily wish your child to participate in the study.

**INFORMED CONSENT FORM**

**Project title:**

Complex integrated multi-component project in the study of developmental dyslexia

Please underline **Yes** or **No** for all statements below (**underline the correct statement**).

I was asked to agree **Yes**  **No**

I read the Information Sheet of the participant in thestudy **Yes** **No**

I was given the opportunity to ask all the questions that were

important to me and my child and to discuss this project **Yes No**

I received satisfactory answers

to all my questions **Yes**  **No**

I received satisfactory answers to all my questions **Yes**  **No**

loudly

I certify with my signature that I was loudly and in detail informed in advance, I asked my questions and received answers to them from: ………………………………………………… ....... .................................................. ....................................................................................

(researcher's name)

I am informed that the conducted research is in accordance with the Declaration of Helsinki (2008) on the ethical principles of medical research in humans and is approved by the Commission on Bioethics at the Institute of Neurobiology, BAS.

If the results of this study are published, the identity of the subject will remain undisclosed.

I agree that my child will voluntarily participate in the study.

I understand that I am free to opt out of my child's participation in the study at any time, without explaining my refusal and without affecting future medical care.

Name, middle name, surname of the examined child:

…………………………………………………………………… ........................ ....................

Name, middle name, surname of parent:

…………………………………………………………………… ........................ ....................

Parent's signature: …………………… ..

The researcher confirms through his/her signature that he/she has provided the subject with the necessary information for the present study.

……………………………

(Researcher's signature)

Date: ……………….
