# Supplementary material for: Speech–Brain Frequency Entrainment of Dyslexia with and without Phonological Deficits
Source: Brain Sci. 2020 Nov 28;10(12):920. doi: 10.3390/brainsci10120920 (PMC7760068; doi:10.3390/brainsci10120920)
Supplement: Supplementary file 1 [file brainsci-10-00920-s001.zip › supp files/Supp file 3 with words pseudowords list.docx]

words

| Крум |  | прав |  | твой |  | ям |
| --- | --- | --- | --- | --- | --- | --- |
| рейс |  | блок |  | крем |  | Спас |
| гост |  | клас |  | лист |  | спя |
| дам |  | щом |  | пак |  | филм |
| цял |  | две |  | през |  | чист |

pseudowords

| дви |  | лест |  | щъм |  | цул |
| --- | --- | --- | --- | --- | --- | --- |
| твей |  | пок |  | блик |  | прув |
| гаст |  | пруз |  | клус |  | руйс |
| дъм |  | Спес |  | им |  | крам |
| фълм |  | чуст |  | Кръм |  | спо |
