# Supplementary material for: Speech–Brain Frequency Entrainment of Dyslexia with and without Phonological Deficits
Source: Brain Sci. 2020 Nov 28;10(12):920. doi: 10.3390/brainsci10120920 (PMC7760068; doi:10.3390/brainsci10120920)

**Table S1**.The group data presented in standard scores.

| 1. Тест DDE-2 | Controls  mean ± s.d. | Dyslexics  mean ±s.d. |
| --- | --- | --- |
| 1.1.Word reading |  |  |
| Accuracy  Time | 106 ± 5.58  132 ± 0.76 | 90.6 ± 4.5  91.7 ± 4.8 |
| 1.2.Nonword reading |  |  |
| Accuracy  Time | 102 ± 4.65  118 ± 0.68 | 88.3 ± 4.4  96.7 ± 4.1 |
| 1.3 Homonyms  Accuracy | 112 ± 4.82 | 98.5 ± 2.4 |
| 1.4 Search for misspellings of words  Accuracy | 112 ± 1.82 | 112 ± 4.45 |
| 1.5 Word writing  Accuracy | 115 ± 6.49 | 85.7 ± 4.4 |
| 1.6 Nonword writing  Accuracy | 104 ± 4.25 | 91.9 ± 3.9 |
| 1.7 Dictation  Accuracy | 112 ± 4.82 | 89.5 ± 3.4 |
| 2. Test battery “Reading abilities” | Controls  mean ± s.d. | Dyslexics  mean ± s.d. |
| 2.1 Phonological tasks  ”without a fist sound- letter” |  |  |
| Correct answers | 9.20 ± 1.87 | 5.52 ± 2.04 |
| Execution time (s) | 34.86 ± 10.5 | 62.5 ± 29.47 |
| 2.2 Phonological task  “without a last syllable” |  |  |
| Correct answers | 8.05 ± 2.08 | 6.15 ± 2.38 |
| Execution time | 37.50 ± 8.8 | 64.84 ± 30.5 |
| 2.3. Text reading |  |  |
| Correct answers | 129.41 ± 3.43 | 119.75 ± 7.88 |
| Execution time (s) | 104.77 ± 29.0 | 191.56 ± 149.55 |
| 2.4 Dictation filling in a missing compound word |  |  |
| Correct sentences | 21.00 ± 5.85 | 10.94 ± 4.51 |
| 3. Raven test | > 98 | > 98 |

**Table S2.** The behaviour parameters of the groups are represented by the percent of the correct answers and reaction time.

| Listening  % / s | Controls | NoPhoDys | PhoDys | | NoPhoDys *vs* Con | | | PhoDys *vs*  Con | | | | | NoPhoDys *vs* PhoDys | | |
| --- | --- | --- | --- | --- | --- | --- | --- | --- | --- | --- | --- | --- | --- | --- | --- |
|  | mean ± s.e. | mean ± s.e. | mean ± s.e. | | *p* | *χ* | | | *p* | *χ* | | | *p* | *χ* |  |
| 1.word |  |  |  |  | | |  |  | | |  |  | |  | |
| success | 83.1±0.73 | 73.1±1.21 | 54.4±1.22 | 0.0001 | | | 38.3 | 0.0001 | | | 135.2 | 0.0001 | | 78.95 | |
| RT | 1416.3±7.06 | 1455.6±12.44 | 1299.6±17.60 | 0.0001 | | | 32.9 | 0.001 | | | 7.7 | 0.0001 | | 35.03 | |
| 2. pseudoword |  |  |  |  | | |  |  | | |  |  | |  | |
| Success | 83.1±0.96 | 75.3±1.74 | 54.4±1.4 | 0.0001 | | | 27.2 | 0.0001 | | | 112.9 | 0.0001 | | 86.02 | |
| RT | 1514.3±8.02 | 1486.0±13. 16 | 1482.7±15.67 | 0.6 | | | 0.2 | 0.1 | | | 2.4 | 0.2 | | 1.64 | |

**Supplemental Figures**

**Figure S1.** Hemispheric asymmetry (left-right hemisphere) for the pseudoword listening condition. Controls are in the first column, NoPhoDys in the second and the PhoDys in the third. Points indicate statistically significant differences (K-W test, bootstrap, p < 0.05). Same format as figure 1.


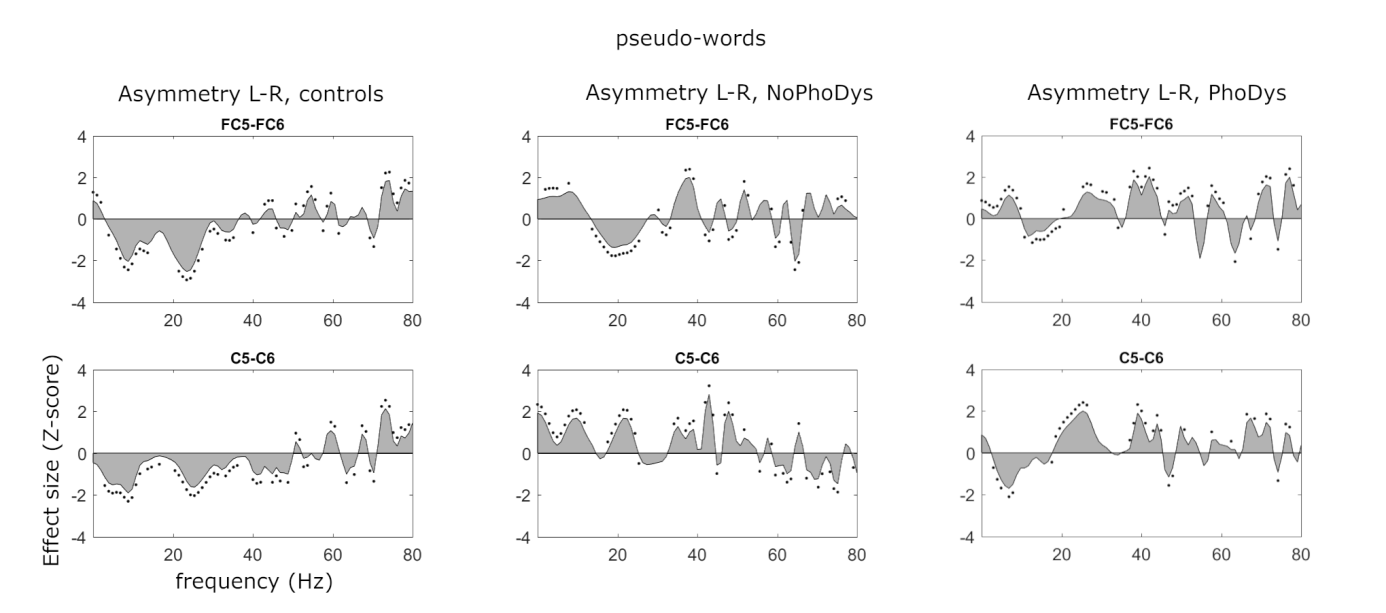


**Figure S2.** Group difference for the pseudo-word listening condition between controls and NoPhoDys, controls and PhoDys, NoPhoDys, and PhoDys. The left column is left IFG and PT; their respective right hemispheric areas are represented in the right column. Points indicate significant differences (K-W test, bootstrap, p < 0.05). Same format as figure 2.


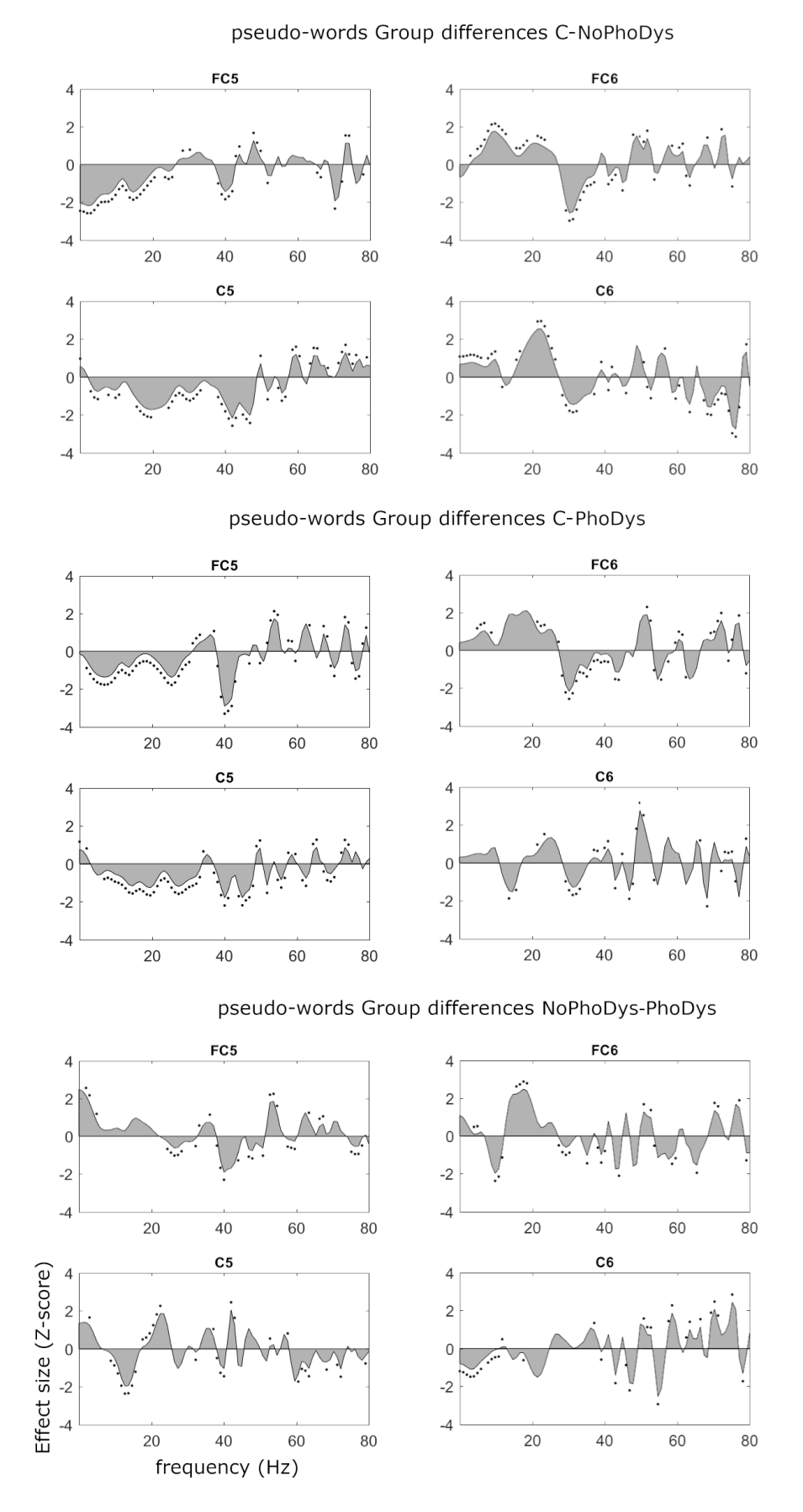

Supplement: Supplementary file 1 [file brainsci-10-00920-s001.zip › supp files/Supplemental Tables S1 S2 Figures S1 S2.docx]
